# The top 100 most-cited papers in pheochromocytomas and paragangliomas: A bibliometric study

**DOI:** 10.3389/fonc.2022.993921

**Published:** 2022-09-16

**Authors:** Sai-Li Duan, Lin Qi, Ming-Hao Li, Long-Fei Liu, Yong Wang, Xiao Guan

**Affiliations:** ^1^ National Clinical Research Center for Geriatric Disorders, Changsha, China; ^2^ Department of General Surgery, Xiangya Hospital Central South University, Changsha, China; ^3^ Department of Urology, Xiangya Hospital, Central South University, Changsha, China

**Keywords:** bibliometric study, top 100, pheochromocytomas, paragangliomas, PPGL

## Abstract

**Background:**

The purpose of this study was to define and analyze the characteristics of the top 100 most-cited articles and reviews on the topic of pheochromocytomas and paragangliomas (PPGLs) by using bibliometric methods.

**Methods:**

We explored the Web of Science Core Collection database to gather the 100 top-cited original articles and reviews of PPGL from 1985 to 20 December 2020. We conducted a bibliometric study to identify the most influential journals, authors, countries, and institutions in the PPGL field.

**Results:**

The 100 top-cited papers were cited a total number of 25,723 times, ranging from 131 to 1,144 (mean, 257.23 ± 173.64). All of these 100 top-cited papers were published between 1999 and 2017, and the number of top-cited papers published before 2008 (1999–2008) was significantly higher than that after 2008 (2009–2017) (p = 0.043). The journal with the highest number of published papers is the *Journal of Clinical Endocrinology & Metabolism* (n = 23). The United States was the most productive country in this topic, which published about half of these publications (n = 51). The National Institutes of Health (NIH) had the largest number of publications (n = 17). Genes or genetics is still the hottest topic in the field of PPGLs.

**Conclusions:**

We defined and analyzed the top 100 most-cited papers in the field of PPGLs by gathering detailed information. These data provided insights into the most influential studies related to PPGL. We hoped to inspire researchers and readers in this field to improve their understanding of PPGL research trends and provide ideas for future research from unique perspectives.

## Introduction

Pheochromocytomas (PHEOs) and paragangliomas (PGLs) are rare neuroendocrine tumors, termed as PPGLs. About 80% to 85% of PPGLs arise from adrenal chromaffin cells (called PHEOs), and the rest originate from extra-adrenal sympathetic or parasympathetic ganglia (called PGLs) (1). A majority of PPGLs are characterized by excessive production of catecholamines (including epinephrine, norepinephrine, and dopamine), and a small proportion of them are biochemically silent (2, 3). Due to the acute release of large amounts of catecholamines into the blood, or long-term adverse effects of catecholamines on the cardiovascular system, patients with PPGLs tend to have a high risk of cardiovascular events (4). The typical symptoms of patients with PPGLs are recurring episodes of hypertension, palpitations, etc. It was reported that about 0.1% to 1% of hypertension was due to PPGLs (3, 5). Particularly, all PPGLs have the potential to metastasize, and the 5-year survival rate of patients with metastatic PPGLs was less than 50% (6). Therefore, there is no doubt that PPGLs are a great threat to human health. Researchers need to pay more attention to the field of PPGLs.

Paul Otlet first introduced bibliometric analysis in 1934, which referred to a tool to identify the key studies and aimed to evaluate the academic influence of a publication or a country in a certain topic or field (7). Bibliometric analysis can provide an external assessment of the quality, influence, and prestige factors of research and examine the development of a particular research area. By using robust academic quality standards to explore published research results, bibliometric analysis is valuable in identifying research trends and cultivating future research ideas (8). Citation analysis could provide comprehensive information on cited papers, which is one of the most important analysis methods in bibliometric analysis (9). The citation frequency, in a particular perspective, can indicate the influence of an article in the discipline, and the research direction of this article can be regarded as the development direction of the field in a certain period. Citation analysis studies have been widely used to explore the research trends in various research fields, such as microRNA (10), lncRNA (11), diabetes (12), and cancer (13).

Considering the continuous growth of PPGL research results, it is imperative to use quantitative methods to evaluate and analyze existing research. However, no bibliometric analysis has been applied to the top 100 most-cited papers in PPGL. We conducted a bibliometric study by determining and describing the top 100 most-cited papers in PPGL and aimed to evaluate the relevant factors of its successful citation, which can help us to understand how the PPGL-related studies have been developed and expanded and possibly benefit the researchers to carry out subsequent research from different perspectives as well as the scientific research cooperation. ^1^

## Methods

### Search strategy

We searched the Web of Science (WOS) Core Collection database to gather studies of PPGLs on 20 December 2020. The following search strategies were used: TI = (“Pheochromocytoma*” OR “paraganglioma*” OR “PPGL*”) and language = English. The publishing year was set from 1985 to 2020.

### Inclusion criteria

**Figure 1:**
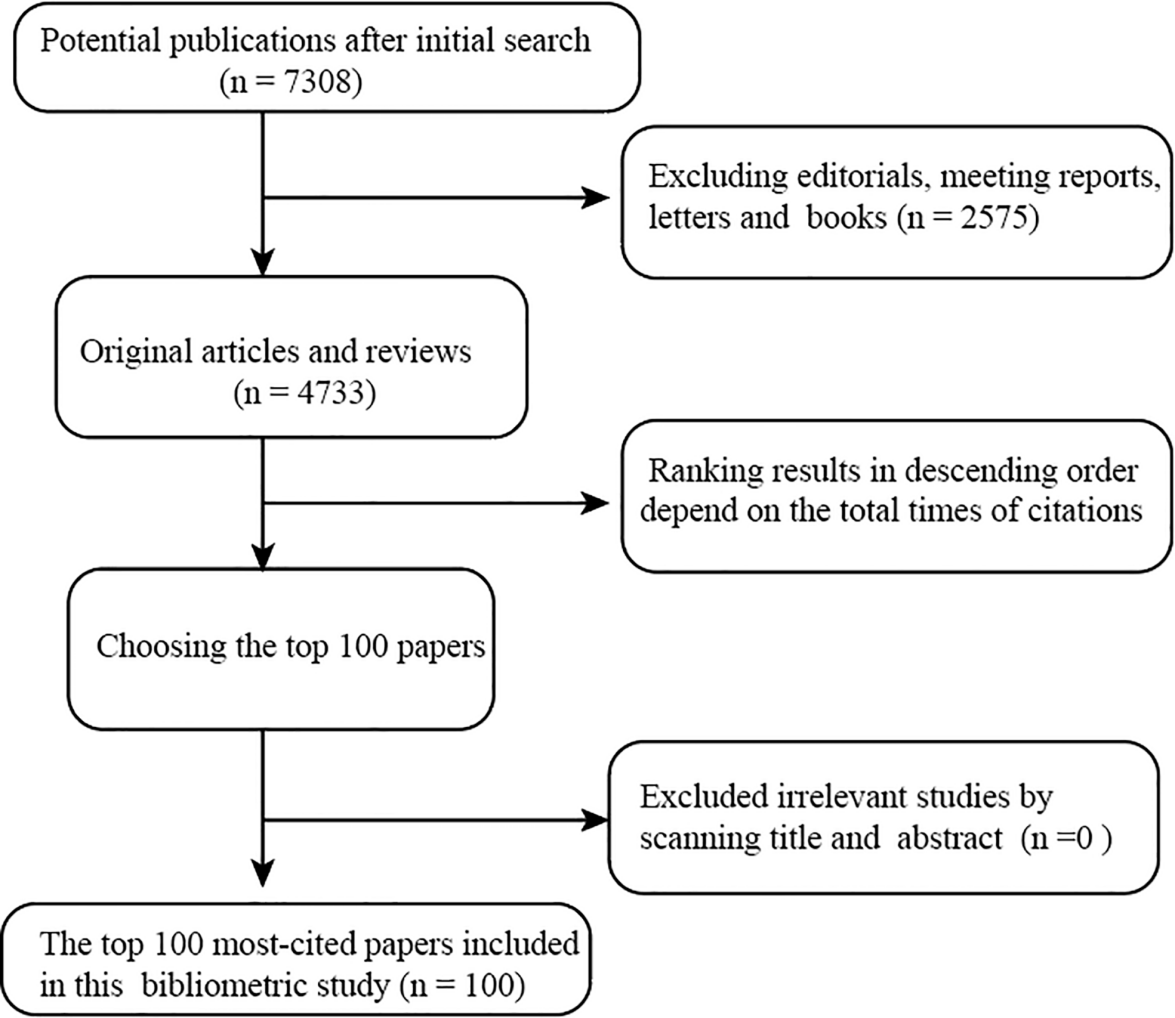
The initial search process in Web of Science (WOS).

Only original articles and reviews were included. Editorials, meeting reports, letters, and books were excluded. The selection results were listed in descending order depending on the total times of citations. We chose the top 100 most-cited papers after reading the title and abstract. The primary selection process is shown in **Figure 1**.

### Data extraction

Two authors (Sai-Li Duan and Yong Wang) independently collected the data, and a third researcher (Minghao Li) was consulted to deal with discrepancies. The following information was collected: first author, corresponding author, article title, journal name, publication year, total cited times, cited times per year, document type, WOS categories, keywords, journal impact factor (IF) 2019 (IF 2019), average impact factor in 5 years (5-year IF), and the addresses of the corresponding author. The first one was selected if there had more than one corresponding author or affiliation.

### Statistical analysis

We used the IBM SPSS Statistics for Windows, version 24.0, software to perform correlation analysis. Correlation analysis was used to evaluate the relationship between the different terms. p < 0.05 (two-tailed) was considered statistically significant.

## Results

### Citations

The top 100 most-cited papers are listed in **Table S1** in descending order depending on the number of total cited times. The 100 top-cited papers were cited a total of 25,723 times with a median citation number of 200.50, ranging from 131 to 1,144 (mean, 257.23 ± 173.64). The average cited times per year of the 100 papers ranged from 6.60 to 135.67, with a median number of 15.50 (mean, 20.41 ± 16.94). The average cited times per year of the 100 papers was correlated with the total cited times (r = 0.692, p < 0.01). The most frequently cited paper was published in *Science* in 2000, which indicated that *SDHD* played an important role in the pathogenesis of hereditary paraganglioma (14) (**Table S1**).

### Publication year

**Figure 2:**
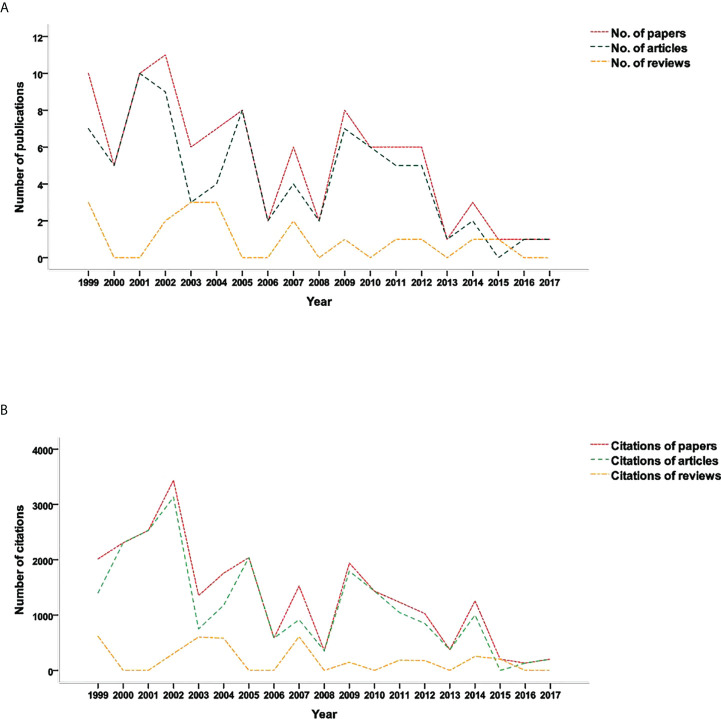
Citations analysis on publication year. **(A)** The relation between publication year and number of publications. **(B)** The relation between publication year and number of citations.

All of these 100 top-cited papers were published between 1999 and 2017. The highest number of top-cited papers was published in 2002 (n = 11), followed by 1999 and 2001 (n = 10). Before 2008 (1999–2008), the number of top-cited papers published was significantly higher than that after 2008 (2009–2017) (p = 0.043). The number of publications of original articles was usually higher than that of reviews (**Figure 2A**), as well as the citation times of original articles and reviews (**Figure 2B**).

### Journals

All of the 100 top-cited papers were published in 50 kinds of journals. Journals that published over two papers (≥3) are listed in **Table 1**. The journal with the highest number of published papers is the *Journal of Clinical Endocrinology & Metabolism* (*JCEM*) (n = 23), followed by the *New England Journal of Medicine* (n = 4) and *Endocrine-Related Cancer* (n = 4). The IF among these 50 journals ranged from 1.585 to 74.699. Total number of citations (r = 0.438, p < 0.01) and average citations (r = 0.337, p < 0.01) were significantly correlated with IF (**Table 1**).

**Table 1:** Journals that published over two papers in the top 100 most-cited papers.

Journal	Number of published papers	Total number of citations	Average citation	IF 2019	5-year IF
*Journal of Clinical Endocrinology & Metabolism*	23	5,528	240.35	5.399	5.879
*New England Journal of Medicine*	4	1,587	396.75	74.699	72.098
*Endocrine-Related Cancer*	4	764	191.00	4.8	5.129
*American Journal of Human Genetics*	3	1,311	437.00	10.502	10.344
*Cancer Cell*	3	966	322.00	26.602	30.237
*Human Molecular Genetics*	3	803	267.67	5.101	5.362
*JAMA—Journal of the American Medical Association*	3	1,542	514.00	45.54	47.677
*Nature Genetics*	3	1,258	419.33	27.605	30.334
*Radiology*	3	658	219.33	7.931	8.237

IF, impact factor.

### Authors

A total of 17 authors who published at least two top-cited papers as the first author are listed in **Table 2**. G. Eisenhofer is the author with the largest number of publications (n = 8). N. Burnichon and H. J. L. M. Timmers both contributed 4 papers. Four authors published three papers, and 10 authors published two papers (**Table 2**). Nineteen corresponding authors who published at least two top-cited papers are listed in **Table 2**. K. Pacak had published the highest number of papers (n = 8), followed by G. Eisenhofer (n = 7) and A. P. Gimenez-Roqueplo (n = 7) (**Table 2**).

**Table 2:** Authors who contributed at least 2 papers to the top 100 most-cited papers list.

First author	Number of top-cited papers	Total cited times	Average citations per paper	Corresponding author	Number of top-cited papers	Total cited times	Average citations per paper
Eisenhofer, G	8	1,537	192.125	Pacak, K	8	1,512	189
Burnichon, N	4	1,008	252	Eisenhofer, G	7	1,397	199.571
Timmers, HJLM	4	806	201.5	Gimenez-Roqueplo, AP	7	1,670	238.571
Amar, L	3	884	294.667	Baysal, BE	3	1,557	519
Baysal, BE	3	1,557	519	Dahia, PLM	3	837	279
Gimenez-Roqueplo, AP	3	623	207.667	Neumann, HPH	3	1,755	585
Pacak, K	3	800	266.667	Plouin, PF	3	480	160
Bayley, JP	2	317	158.5	Young, WF	3	725	241.667
Carney, JA	2	468	234	Burnichon, N	2	615	307.5
Dahia, PLM	2	576	288	Carney, JA	2	468	234
Favier, J	2	367	183.5	Cascon, A	2	470	235
Ilias, I	2	355	177.5	Eng, C	2	483	241.5
Lenders, JWM	2	1,511	755.5	Favier, J	2	537	268.5
Mannelli, M	2	333	166.5	Lenders, JWM	2	1511	755.5
Neumann, HPH	2	1,524	762	Mannelli, M	2	333	166.5
Plouin, PF	2	269	134.5	Taschner, PEM	2	279	139.5
Walther, MM	2	343	171.5	Timmers, HJLM	2	471	235.5
				Tischler, AS	2	550	275
				Walther, MM	2	343	171.5

### Countries and institutions

All of the top 100 most-cited papers originated from 13 countries, as shown in **Table 3**. The United States and France were the most productive countries in this topic; about half of these publications (n = 51) were from the United States, and 17% (n = 17) were from France. The Netherlands ranked third with eight papers, followed by Germany (n = 7), Italy (n = 4), and Spain (n = 4). A total of 18 institutions have published more than one paper (≥2), as listed in **Table 4**. The National Institutes of Health (NIH)—USA NIH had the largest number of publications (n = 17), followed by APHP Hop Europeen Georges Pompidou (n = 11) and Mayo Clinic Mayo Clin and Mayo Fdn (n = 6) (**Table 4**).

**Table 3:** Countries that published at least one paper in the top 100 most-cited papers.

Countries	Number of publications	Total citations	Average citations per paper
USA	51	12,914	253.2157
France	17	3,920	230.5882
Netherlands	8	1,954	244.25
Germany	7	2,990	427.1429
Italy	4	640	160
Spain	4	966	241.5
Austria	2	323	161.5
Australia	2	539	269.5
England	1	768	768
Greece	1	196	196
South Korea	1	176	176
Sweden	1	187	187
Switzerland	1	150	150
Total	100	25,723	257.23

**Table 4:** Institutions that published at least two papers in the top 100 most-cited papers.

Institutions	Number of papers	Total citation times	Citations per paper
National Institutes of Health (NIH)—USA NIH	17	3,250	191.176
APHP Hop Europeen Georges Pompidou	11	2,627	238.818
Mayo Clinic Mayo Clin and Mayo Fdn	6	1,333	222.167
Leiden University Leiden Univ, Med Ctr	4	600	150.000
University of Freiburg Univ Freiburg	4	1,993	498.250
University of Texas System University	4	1,013	253.250
Pennsylvania Commonwealth System of Higher Education (PCSHE) University of Pittsburgh Univ Pittsburgh, Med Ctr	3	1,557	519.000
Spanish National Cancer Research Centre	3	827	275.667
Harvard University	3	691	230.333
Institut National de la Sante et de la Recherche Medicale (Inserm) Universite de Paris INSERM	3	715	238.333
Erasmus University	2	412	206.000
James Cancer Hospital and Solove Research Institute Ohio State University Ohio State Univ	2	483	241.500
Radboud University Nijmegen Radboud Univ Nijmegen, Med Ctr	2	942	471.000
Royal North Shore Hospital Royal N Shore Hosp	2	539	269.500
Technische Universitat Dresden Univ Dresden	2	331	165.500
Tufts Medical Center Tufts Med Ctr	2	550	275.000
United States Department of Defense Armed Forces Inst Pathol	2	495	247.500
University of Florence Univ Florence	2	333	166.500

### Type of documents and publications

Among the 100 top-cited papers, 82 were original articles, and 18 were reviews. There was no significant difference in total citations (p = 0.159) and average citations per year (p = 0.341) between original articles and reviews. However, it needs to be emphasized that the average number of total citations for original articles (n = 268.71) was higher than that of reviews (n = 204.94).

A total of 63 of the 100 top-cited papers chose the publication type of open access (OA), while the remaining 37 papers were published as non-open access (non-OA). The type of publication was not significant with the number of citations (p = 0.406), but the average number of total citations of non-OA papers (n = 276.16) was higher than that of OA papers (n = 246.11).

### Web of Science categories

The 100 top-cited publications were divided into 24 WOS categories based on their respective research topics. The majority of the publications were categorized into “endocrinology and metabolism” (n =34) and “oncology” (n = 21). These papers were also classified into other fields, such as “genetics and heredity” (n = 16), “medicine, general and internal” (n = 11), “radiology, nuclear medicine and medical imaging” (n = 6), and other categories (**Table 5**).

**Table 5:** WOS categories in the top 100 most-cited papers.

WOS categories	Times
Endocrinology and metabolism	34
Oncology	21
Genetics and heredity	16
Medicine, general and internal	11
Radiology, nuclear medicine and medical imaging	6
Biochemistry and molecular biology	4
Cell biology	3
Multidisciplinary sciences	3
Pathology	3
Surgery	3
Urology and nephrology	3
Medical laboratory technology	2
Peripheral vascular disease	1
Anesthesiology	1
Biotechnology and applied microbiology	1
Cardiac and cardiovascular systems	1
Dentistry, oral surgery and medicine	1
Gastroenterology and hepatology	1
Medicine, research and experimental	1
Neurosciences	1
Otorhinolaryngology	1
Peripheral vascular disease	1
Respiratory system	1
Toxicology	1

WOS, Web of Science.

### Keywords and research trends

The 100 papers had a total of 842 keywords (including repetition), about an average of eight keywords per paper. Keywords that occur more than five times (≥6) are listed in **Table 6**. We could see that the top 5 keywords listed were *gene-mutations*, *complex-II gene*, *diagnosis*, *MIBG*, and *VHL* (different keywords representing the same meaning were treated as the same keyword, such as tumor and tumors, gene-mutations and germline mutations, and multiple endocrine neoplasms and MEN). The keywords listed in **Table 6** could represent the research hotspots in this field of PPGL from a unique perspective.

**Table 6:** Keywords that appeared at least five times in the top 100 most-cited papers.

Keywords	Numbers	Keywords	Numbers
Gene-mutations	41	Familial pheochromocytoma	7
Diagnosis	30	MEN	7
Complex-II gene	26	Sporadic pheochromocytoma	7
MIBG	23	Susceptibility	7
VHL	20	Cancer	6
Malignant pheochromocytoma	14	Carotid-body tumors	6
Succinate-dehydrogenase	14	Expression	6
MEN-2	11	Head	6
PET	11	Hereditary paragangliomas	6
Tumors	11	Nonchromaffin paragangliomas	6
Management	10	SDHB	6
Localization	9		
Tumor-suppressor gene	9		
Benign	8		
Gene	8		
RET	8		
Catecholamines	7		

**Figure 3:**
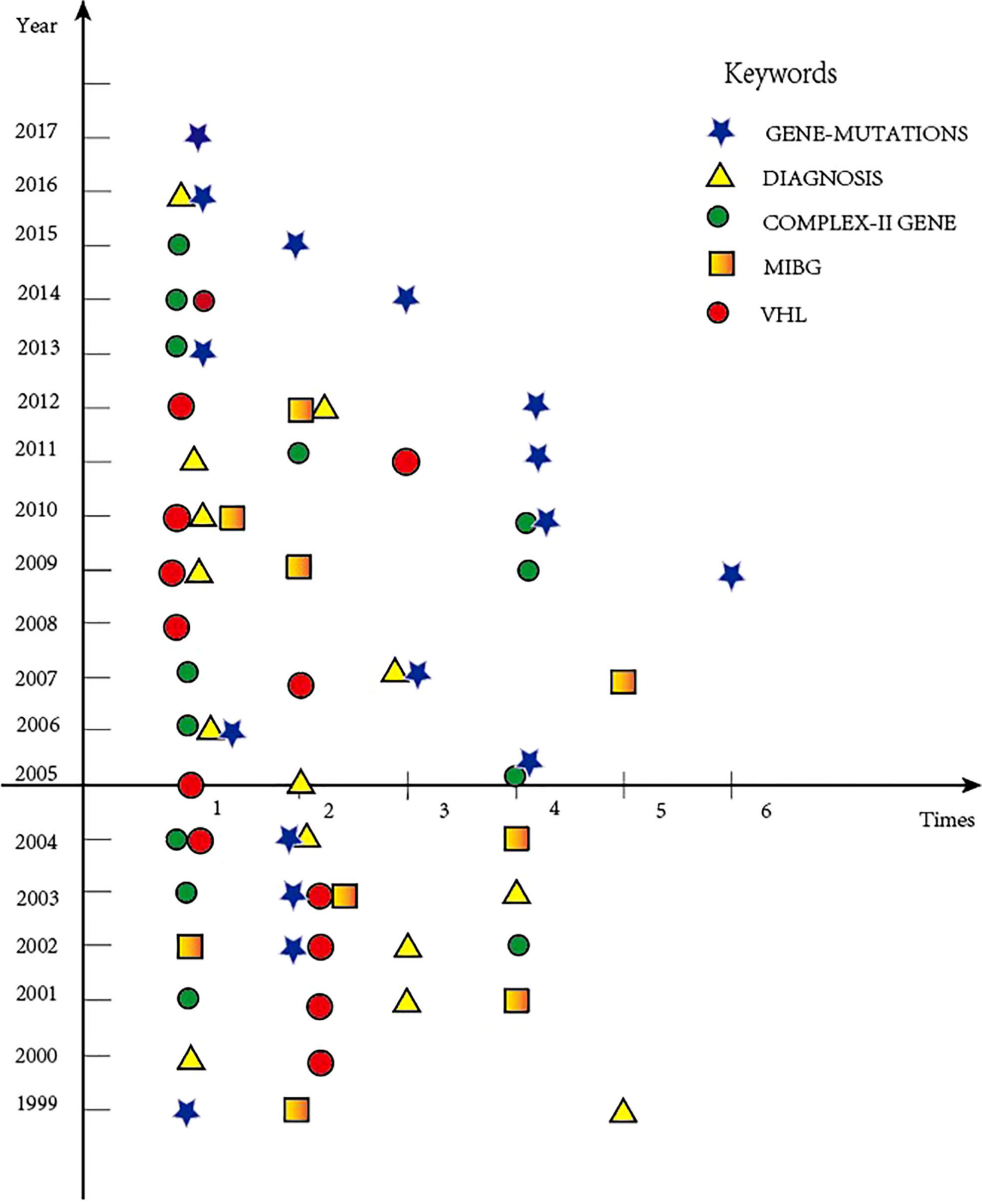
The relationship between top 5 keywords and year.

In addition, we marked the changes in these five keywords each year to describe the research trends (**Figure 3**). It can be seen that gene-mutations gradually develop and expand in the field of PPGL in 2002 and reach the peak of citation around 2009. The citation peak of the keyword *MIBG* was around 2007. *Complex-II gene*, *VHL*, and *diagnosis* have always been an integral part of the PPGL research field.

## Discussion

In this bibliometric study, we selected the top 100 most-cited original articles and reviews in the field of PHEOs and PGLs from the WOS Core English database from 1985 to 20 December 2020. We are now discussing the characteristics of the top 100 most-cited papers and some of our previous results to review the development history and prospects of PPGLs.

The number of citations has still been used as a reliable objective marker of the quality and impact of a paper; it is variable in different subdisciplines and depends on the size of the field of study. In our data, the number of citations times of the top 100 cited original articles and reviews is 131 to 1,144, with a total cited of 25,723; it is lower than some hot research fields such as lncRNAs (15) and neuro-interventional (16) but higher than that of a meta-analysis of radiology (17), suggesting that PPGL research worldwide was in a tepid period. The PPGL research field was still not prominent enough in the world. As we mentioned above, PPGL is an important cause of secondary hypertension. Hypertension and cardiovascular as well as cerebrovascular diseases caused by PPGL are extremely harmful to human health. Therefore, we need to increase investment in this field.

All papers were published between 1999 and 2017. The number of citations of papers before 2008 (1999–2008) was significantly higher than that after 2008 (2009–2017) (p = 0.043). Articles published in the recent 3 years were not included. This indicates that time was a very important factor for citations, and even the latest important achievements have difficulty obtaining high citations within a short period of time.

The 100 papers were published in a total of 50 journals, among which nine journals had published more than two papers (≥3). The IF of these journals was from 1.585 to 74.699. The *JCEM* had the largest number of published PPGL research papers (n = 23), with a total of 23 papers cited 5528 times, each cited a mean of 240.35 times, which was far higher than *JCEM* IF in 5 years (5.879). In addition, the lowest IF of the 50 journals was 1.585, suggesting that even the low-IF journal could also produce high-citation papers. It also indicated that we should pay more attention to the quality of papers rather than the “IF” of the journal. What matters is how much the paper made practical contributions to the field, which is the key to being cited, although the average number of citations was significantly correlated with the IF of the journal.

The United States is the country with the most published papers in the PPGL research field worldwide, from which more than half (51) are produced. France, New Zealand, and Germany made important contributions to this field. NIH (n = 17) and APHP (n = 11) were institutions that published the most PPGL studies. This is consistent with the countries.

Among the 100 papers, 82% (n = 82) were original articles, and 18% (n = 18) were reviews. The total number of citations and the average number of citations were independent of literature type and publication mode. WOS categories with the most frequency was “endocrinology and metabolism” (n = 34), followed by “oncology” (n = 21) and “genetics and heredity” (n = 16).

High-citation papers have research hotpots in a specific field, and the keywords were an important symbol of these papers. These keywords were the top 5 keywords in this topic, including *gene-mutations*, *complex-II gene*, *diagnosis*, *MIBG*, and *VHL*. Among these, *gene-mutations*, *complex-II gene*, and *VHL* belong to a bigger category called “*gene*”, which could be considered the most frequent keyword in the field of PPGL. We could see that the research hotspots in PPGL would change over time, and this might be the same with other fields as well. If the peak of keywords has passed and no longer appears again, this might indicate that the problem might have been answered. Instead, it could be considered that this problem has not been solved if it was cited frequently in recent years. With more and more papers published, problems might be solved, but the keywords will appear at the same time, prompting us to keep moving forward.

Genes or genetics is still the hottest topic in the field of PPGLs. As a rare disease, the low incidence rate has become an obstacle to researchers of PPGLs. Our results showed that Chinese or Indian scholars were not prominent in this field. However, China or India has a large population, which provides a prior condition for studies of PPGLs, which suggests that the top research institutions may have potential cooperation with institutions in these countries. However, it was reported that ethnic differences may lead to great genetic heterogeneity (18), which will require international cooperation for better development in this field.

Our research also found that top research institutions have a large number of research results in the field of PPGLs, which enlightens us to pay close attention to these institutions when seeking cooperation. When we need to search for the latest findings in the PPGLs field, we can take the top institutions, authors, or journals in the results of this study as the search objects.

Unfortunately, the present study still has some unavoidable limitations. The first and most significant one is that we selected these papers only depending on their cited frequency. The latest important achievements have difficulty obtaining high citations within a short period of time to be included, and old papers may become less cited with time as they become common knowledge or irrelevant (19). Second, we only used the WOS database for analysis, and we included only studies published in English. The number of citations may be inconsistent between different databases and languages. Third, the citations for the 100 most-cited papers change over time, and even those at the end of the 100 are replaced at different time points. Fourth, although we have been very careful in the search process, we still may have missed some PPGL studies because the keywords were not mentioned in the paper.

Despite these limitations, our study is the first to apply the bibliometric method to analyze the 100 papers with the highest number of citations in PPGL by collecting information including the number of citations, year of publication, journals, authors, countries and institutions, document type, publishing type, and WOS categories. The most influential countries, institutions, journals, authors, and WOS categories commonly used in PPGL were identified, which has significantly contributed toward advancements in this specialty. We believe that as the first citation analysis in PPGL, our conclusions will contribute to the understanding of trends and classic publications on this topic. This may be helpful to determine future research trends and cooperation between countries and institutions worldwide. Furthermore, this bibliometric study may be beneficial for the education of medical students.

## Conclusions

We defined and analyzed the top 100 most-cited papers in the field of PPGLs by gathering detailed information. These data provide insights into the most influential studies related to PPGL. We hope to inspire researchers and readers in this field to improve their understanding of PPGL research trends and provide ideas for future research from unique perspectives.

## Data availability statement

The original contributions presented in the study are included in the article/**Supplementary Material**. Further inquiries can be directed to the corresponding authors.

## Author contributions

S-LD, YW and XG conceived and planned the study design. S-LD, LQ, M-HL, L-FL, YW and XG collected formal resources and wrote the original draft. YW and XG provided critical revisions and contributed to the editing of the paper. All authors contributed to the article and approved the submitted version.

## Funding

This work was supported by the National Natural Science Foundation of China (No. 81902727 to XG) and the National Science Foundation of Hunan Province (No. 2022JJ40781 to XG).

## Conflict of interest

The authors declare that the research was conducted in the absence of any commercial or financial relationships that could be construed as a potential conflict of interest.

## Publisher’s note

All claims expressed in this article are solely those of the authors and do not necessarily represent those of their affiliated organizations, or those of the publisher, the editors and the reviewers. Any product that may be evaluated in this article, or claim that may be made by its manufacturer, is not guaranteed or endorsed by the publisher.

## Footnotes

1 The authors are accountable for all aspects of the work in ensuring that questions related to the accuracy or integrity of any part of the work are appropriately investigated and resolved.

## Abbreviations

PHEOs, pheochromocytomas; PGLs, paragangliomas; WOS, Web of Science; OA, open access; non-OA, non-open access.

## References

[1] Wang Y Li M Deng H Pang Y Liu L Guan X. The systems of metastatic potential prediction in pheochromocytoma and paraganglioma. Am J Cancer Res (2020) 10:769–80. PMC7136918 32266090

[2] Erlic Z Kurlbaum M Deutschbein T Nolting S Prejbisz A Timmers H. Metabolic impact of pheochromocytoma/paraganglioma: Targeted metabolomics in patients before and after tumor removal. Eur J Endocrinol (2019) 181:647–57. doi: 10.1530/EJE-19-0589 31614337

[3] Pappachan JM Tun NN Arunagirinathan G Sodi R Hanna FWF. Pheochromocytomas and hypertension. Curr Hypertens Rep (2018) 20:3. doi: 10.1007/s11906-018-0804-z 29356966

[4] Petrak O Rosa J Holaj R Strauch B Kratka Z Kvasnicka J. Catecholamine phenotype, and target organ damage in Pheochromocytoma/Paraganglioma. J Clin Endocrinol Metab (2019) 104:5170–80. doi: 10.1210/jc.2018-02644 31009053

[5] Lenders JW Duh QY Eisenhofer G Gimenez-Roqueplo AP Grebe SK Murad MH. Pheochromocytoma and paraganglioma: An endocrine society clinical practice guideline. J Clin Endocrinol Metab (2014) 99:1915–42. doi: 10.1210/jc.2014-1498 24893135

[6] Hescot S Leboulleux S Amar L Vezzosi D Borget I Bournaud-Salinas C. One-year progression-free survival of therapy-naive patients with malignant pheochromocytoma and paraganglioma. J Clin Endocrinol Metab (2013) 98:4006–12. doi: 10.1210/jc.2013-1907 23884775

[7] Rousseau R. Library science: Forgotten founder of bibliometrics. Nature (2014) 510:218. doi: 10.1038/510218e 24919911

[8] Zhang Y Xiong Y Cai Y Zheng L Zhang Y. The 100 top-cited studies on neuropsychology: A bibliometric analysis. Front Psychol (2020) 11:550716. doi: 10.3389/fpsyg.2020.550716 33329180 PMC7734023

[9] Shadgan B Roig M Hajghanbari B Reid WD. Top-cited articles in rehabilitation. Arch Phys Med Rehabil (2010) 91:806–15. doi: 10.1016/j.apmr.2010.01.011 20434622

[10] Casey MC Kerin MJ Brown JA Sweeney KJ. Evolution of a research field-a micro (RNA) example. PeerJ (2015) 3:e829. doi: 10.7717/peerj.829 25802804 PMC4369334

[11] Chen X Shi Y Zhou K Yu S Cai W Ying M. A bibliometric analysis of long non-coding RNA and chemotherapeutic resistance research. Oncotarget (2019) 10:3267–75. doi: 10.18632/oncotarget.26938 PMC6524938 31143372

[12] Yang Q Guo N Zhou Y Chen J Wei Q Han M. The role of tumor-associated macrophages (TAMs) in tumor progression and relevant advance in targeted therapy. Acta Pharm Sin B (2020) 10:2156–70. doi: 10.1016/j.apsb.2020.04.004 PMC7714989 33304783

[13] Cabral BP da Graca Derengowski Fonseca M Mota FB. The recent landscape of cancer research worldwide: A bibliometric and network analysis. Oncotarget (2018) 9:30474–84. doi: 10.18632/oncotarget.25730 PMC6078146 30093962

[14] Baysal BE Ferrell RE Willett-Brozick JE Lawrence EC Myssiorek D Bosch A. Mutations in SDHD, a mitochondrial complex II gene, in hereditary paraganglioma. Science (2000) 287:848–51. doi: 10.1126/science.287.5454.848 10657297

[15] Peng MS Chen CC Wang J Zheng YL Guo JB Song G. The top 100 most-cited papers in long non-coding RNAs: A bibliometric study. Cancer Biol Ther (2020) 22(1):40–54. doi: 10.1080/15384047.2020.1844116 33315532 PMC7836983

[16] Kim ES Yoon DY Kim HJ Jeon HJ Lee JY Cho BM. Citation classics in neurointerventional research: A bibliometric analysis of the 100 most cited articles. J neurointerv Surg (2017) 9:508–11. doi: 10.1136/neurintsurg-2016-012399 27127230

[17] Yaxley KL To MS. The 100 top-cited meta-analyses of diagnostic accuracy in radiology journals: A bibliometric analysis. Insights Imaging (2020) 11:123. doi: 10.1186/s13244-020-00936-w 33226503 PMC7683640

[18] Jiang J Zhang J Pang Y Bechmann N Li M Monteagudo M. Sino-European differences in the genetic landscape and clinical presentation of pheochromocytoma and paraganglioma. J Clin Endocrinol Metab (2020) 105(10). doi: 10.1210/clinem/dgaa502 32750708

[19] Nov E Moisseiev E. The top 100 most-cited papers on intravitreal injections: A bibliographic perspective. Clin Ophthalmol (2020) 14:2757–72. doi: 10.2147/OPTH.S267617 PMC7519813 33061258

