# Supplementary material for: The top 100 most-cited papers in pheochromocytomas and paragangliomas: A bibliometric study
Source: Front Oncol. 2022 Sep 16;12:993921. doi: 10.3389/fonc.2022.993921 (PMC9523535; doi:10.3389/fonc.2022.993921)
Supplement: Supplementary file 1 [file Table_1.docx]

**Table 1. The top 100 most-cited papers in PPGLs filed.**

| Rank | First author | Paper Title | Publication  Year | Total Cited times | Cited times per year |
| --- | --- | --- | --- | --- | --- |
| 1 | Baysal, BE | Mutations in SDHD, a mitochondrial complex II gene, in hereditary paraganglioma | 2000 | 1144 | 57.20 |
| 2 | Neumann, HPH | Germ-line mutations in nonsyndromic pheochromocytoma. | 2002 | 910 | 50.56 |
| 3 | Lenders, JWM | Pheochromocytoma and Paraganglioma: An Endocrine Society Clinical Practice Guideline | 2014 | 814 | 135.67 |
| 4 | Astuti, D | Gene mutations in the succinate dehydrogenase subunit SDHB cause susceptibility to familial pheochromocytoma and to familial paraganglioma | 2001 | 768 | 40.42 |
| 5 | Lenders, JWM | Biochemical diagnosis of pheochromocytoma - Which test is best? | 2002 | 697 | 38.72 |
| 6 | Niemann, S | Mutations in SDHC cause autosomal dominant paraganglioma, type 3 | 2000 | 666 | 33.30 |
| 7 | Neumann, HPH | Distinct clinical features of paraganglioma syndromes associated with SDHB and SDHD gene mutations | 2004 | 614 | 38.38 |
| 8 | Hao, HX | SDH5, a Gene Required for Flavination of Succinate Dehydrogenase, Is Mutated in Paraganglioma | 2009 | 481 | 43.73 |
| 9 | Burnichon, N | SDHA is a tumor suppressor gene causing paraganglioma | 2010 | 437 | 43.70 |
| 10 | Amar, L | Genetic testing in pheochromocytoma or functional paraganglioma | 2005 | 429 | 28.60 |
| 11 | Pacak, K | Pheochromocytoma: recommendations for clinical practice from the First International Symposium | 2007 | 415 | 31.92 |
| 12 | Lee, S | Neuronal apoptosis linked to EgIN3 prolyl hydroxylase and familial pheochromocytoma genes: Developmental culling and cancer | 2005 | 387 | 25.80 |
| 13 | Benn, DE | Clinical presentation and penetrance of pheochromocytoma/paraganglioma syndromes | 2006 | 383 | 27.36 |
| 14 | Letouze, E | SDH Mutations Establish a Hypermethylator Phenotype in Paraganglioma | 2013 | 374 | 53.43 |
| 15 | Thompson, LDR | Pheochromocytoma of the adrenal gland scaled score (PASS) to separate benign from malignant neoplasms - A clinicopathologic and immunophenotypic study of 100 cases | 2002 | 337 | 18.72 |
| 16 | Comino-Mendez, I | Exome sequencing identifies MAX mutations as a cause of hereditary pheochromocytoma | 2011 | 331 | 36.78 |
| 17 | van Nederveen, FH | An immunohistochemical procedure to detect patients with paraganglioma and phaeochromocytoma with germline SDHB, SDHC, or SDHD gene mutations: a retrospective and prospective analysis | 2009 | 329 | 29.91 |
| 18 | Dahia, PLM | A HIF1 alpha regulatory loop links hypoxia and mitochondrial signals in pheochromocytomas | 2005 | 320 | 21.33 |
| 19 | Bravo, EL | Pheochromocytoma: State-of-the-art and future prospects | 2003 | 306 | 18.00 |
| 20 | Erickson, D | Benign paragangliomas: Clinical presentation and treatment outcomes in 236 patients | 2001 | 299 | 15.74 |
| 21 | Sawka, AM | A comparison of biochemical tests for pheochromocytoma: Measurement of fractionated plasma metanephrines compared with the combination of 24-hour urinary metanephrines and catecholamines | 2003 | 295 | 17.35 |
| 22 | Goldstein, RE | Clinical experience over 48 years with pheochromocytoma | 1999 | 285 | 13.57 |
| 23 | Gimenez-Roqueplo, AP | The R22X mutation of the SDHD gene in hereditary paraganglioma abolishes the enzymatic activity of complex II in the mitochondrial respiratory chain and activates the hypoxia pathway | 2001 | 282 | 14.84 |
| 24 | Eisenhofer, G | Biochemical diagnosis of pheochromocytoma: How to distinguish true- from false-positive test results | 2003 | 278 | 16.35 |
| 25 | Carney, JA | Gastric stromal sarcoma, pulmonary chondroma, and extra-adrenal paraganglioma (Carney triad): Natural history, adrenocortical component, and possible familial occurrence | 1999 | 267 | 12.71 |
| 26 | Chen, H | The North American Neuroendocrine Tumor Society Consensus Guideline for the Diagnosis and Management of Neuroendocrine Tumors Pheochromocytoma, Paraganglioma, and Medullary Thyroid Cancer | 2010 | 263 | 26.30 |
| 27 | Qin, YJ | Germline mutations in TMEM127 confer susceptibility to pheochromocytoma | 2010 | 261 | 26.10 |
| 28 | Vanharanta, S | Early-onset renal cell carcinoma as a novel extra paraganglial component of SDHB-associated heritable paraganglioma | 2004 | 261 | 16.31 |
| 29 | Dahia, PLM | Pheochromocytoma and paraganglioma pathogenesis: learning from genetic heterogeneity | 2014 | 256 | 42.67 |
| 30 | Pacak, K | Approach to the patient - Preoperative management of the pheochromocytoma patient | 2007 | 250 | 19.23 |
| 31 | Baysal, BE | Prevalence of SDHB, SDHC, and SDHD germline mutations in clinic patients with head and neck paragangliomas | 2002 | 247 | 13.72 |
| 32 | Timmers, HJLM | Comparison of F-18-Fluoro-L-DOPA, F-18-Fluoro-Deoxyglucose, and F-18-Fluorodopamine PET and I-123-MIBG Scintigraphy in the Localization of Pheochromocytoma and Paraganglioma | 2009 | 245 | 22.27 |
| 33 | Amar, L | Succinate dehydrogenase B gene mutations predict survival in patients with malignant pheochromocytomas or paragangliomas | 2007 | 244 | 18.77 |
| 34 | Eisenhofer, G | Malignant pheochromocytoma: current status and initiatives for future progress | 2004 | 241 | 15.06 |
| 35 | Hoegerle, S | Pheochromocytomas: Detection with F-18 DOPA whole-body PET - Initial results | 2002 | 238 | 13.22 |
| 36 | Zhuang, ZP | Somatic HIF2A Gain-of-Function Mutations in Paraganglioma with Polycythemia | 2012 | 236 | 29.50 |
| 37 | Schiavi, F | Predictors and prevalence of paraganglioma syndrome associated with mutations of the SDHC gene | 2005 | 231 | 15.40 |
| 38 | Shulkin, BL | Pheochromocytomas: Imaging with 2-[fluorine-18] fluoro-2-deoxy-D-glucose PET | 1999 | 229 | 10.90 |
| 39 | van der Harst, E | [I-123] metaiodobenzylguanidine and [In-111] octreotide uptake in benign and malignant pheochromocytomas | 2001 | 227 | 11.95 |
| 40 | Burnichon, N | The Succinate Dehydrogenase Genetic Testing in a Large Prospective Series of Patients with Paragangliomas | 2009 | 226 | 20.55 |
| 41 | Timmers, HJLM | Superiority of fluorodeoxyglucose positron emission tomography to other functional imaging techniques in the evaluation of metastatic SDHB-associated pheochromocytoma and paraganglioma | 2007 | 226 | 17.38 |
| 42 | Ladroue, C | Brief Report: PHD2 Mutation and Congenital Erythrocytosis with Paraganglioma | 2008 | 222 | 18.50 |
| 43 | Gimm, O | Somatic and occult germ-line mutations in SDHD, a mitochondrial complex II gene, in nonfamilial pheochromocytoma | 2000 | 222 | 11.10 |
| 44 | Eisenhofer, G | Plasma normetanephrine and metanephrine for detecting pheochromocytoma in Von Hippel-Lindau disease and multiple endocrine neoplasia type 2 | 1999 | 219 | 10.43 |
| 45 | Fu, WM | Anti-apoptotic role of telomerase in pheochromocytoma cells | 1999 | 219 | 10.43 |
| 46 | Amar, L | Year of diagnosis, features at presentation, and risk of recurrence in patients with pheochromocytoma or secreting paraganglioma | 2005 | 211 | 14.07 |
| 47 | Fishbein, L | Comprehensive Molecular Characterization of Pheochromocytoma and Paraganglioma | 2017 | 205 | 68.33 |
| 48 | Favier, J | Paraganglioma and phaeochromocytoma: from genetics to personalized medicine | 2015 | 204 | 40.80 |
| 49 | Brouwers, FM | High frequency of SDHB germline mutations in patients with malignant catecholamine-producing paragangliomas: Implications for genetic testing | 2006 | 201 | 14.36 |
| 50 | Carney, JA | Familial paraganglioma and gastric stromal sarcoma: A new syndrome distinct from the Carney triad | 2002 | 201 | 11.17 |
| 51 | Lee, JH | National Cancer Data Base report on malignant paragangliomas of the head and neck | 2002 | 200 | 11.11 |
| 52 | Eisenhofer, G | Pheochromocytomas in von Hippel-Lindau syndrome and multiple endocrine neoplasia type 2 display distinct biochemical and clinical phenotypes | 2001 | 197 | 10.37 |
| 53 | Chrisoulidou, A | The diagnosis and management of malignant phaeochromocytoma and paraganglioma | 2007 | 196 | 15.08 |
| 54 | Timmers, HJLM | Clinical presentations, biochemical phenotypes, and genotype-phenotype correlations in patients with succinate dehydrogenase subunit B-associated pheochromocytomas and paragangliomas | 2007 | 196 | 15.08 |
| 55 | Szolar, DH | Adrenocortical carcinomas and adrenal pheochromocytomas: Mass and enhancement loss evaluation at delayed contrast-enhanced CT | 2005 | 191 | 12.73 |
| 56 | Walther, MM | von Recklinghausen's disease and pheochromocytomas | 1999 | 190 | 9.05 |
| 57 | Castro-Vega, LJ | Germline mutations in FH confer predisposition to malignant pheochromocytomas and paragangliomas | 2014 | 188 | 31.33 |
| 58 | Welander, J | Genetics and clinical characteristics of hereditary pheochromocytomas and paragangliomas | 2011 | 187 | 20.78 |
| 59 | Mannelli, M | Clinically Guided Genetic Screening in a Large Cohort of Italian Patients with Pheochromocytomas and/or Functional or Nonfunctional Paragangliomas | 2009 | 186 | 16.91 |
| 60 | Korpershoek, E | SDHA Immunohistochemistry Detects Germline SDHA Gene Mutations in Apparently Sporadic Paragangliomas and Pheochromocytomas | 2011 | 185 | 20.56 |
| 61 | Gimenez-Roqueplo, AP | An Update on the Genetics of Paraganglioma, Pheochromocytoma, and Associated Hereditary Syndromes | 2012 | 179 | 22.38 |
| 62 | Ilias, I | Current approaches and recommended algorithm for the diagnostic localization of pheochromocytoma | 2004 | 179 | 11.19 |
| 63 | Burnichon, N | Integrative genomic analysis reveals somatic mutations in pheochromocytoma and paraganglioma | 2011 | 178 | 19.78 |
| 64 | Bayley, JP | SDHAF2 mutations in familial and sporadic paraganglioma and phaeochromocytoma | 2010 | 177 | 17.70 |
| 65 | Ayala-Ramirez, M | Clinical Risk Factors for Malignancy and Overall Survival in Patients with Pheochromocytomas and Sympathetic Paragangliomas: Primary Tumor Size and Primary Tumor Location as Prognostic Indicators | 2011 | 176 | 19.56 |
| 66 | Ilias, I | Superiority of 6-[F-18]-fluorodopamine positron emission tomography versus [I-131]-metaiodobenzylguanidine scintigraphy in the localization of metastatic pheochromocytoma | 2003 | 176 | 10.35 |
| 67 | Jang, JH | Protective effects of resveratrol on hydrogen peroxide-induced apoptosis in rat pheochromocytoma (PC12) cells | 2001 | 176 | 9.26 |
| 68 | Eisenhofer, G | Measurements of Plasma Methoxytyramine, Normetanephrine, and Metanephrine as Discriminators of Different Hereditary Forms of Pheochromocytoma | 2011 | 175 | 19.44 |
| 69 | Kim, DSHL | Curcuminoids from Curcuma longa L. (Zingiberaceae) that protect PC12 rat pheochromocytoma and normal human umbilical vein endothelial cells from PA (1-42) insult | 2001 | 172 | 9.05 |
| 70 | Burnichon, N | MAX Mutations Cause Hereditary and Sporadic Pheochromocytoma and Paraganglioma | 2012 | 167 | 20.88 |
| 71 | Bryant, J | Pheochromocytoma: The expanding genetic differential diagnosis | 2003 | 167 | 9.82 |
| 72 | Baysal, BE | Hereditary paraganglioma target's diverse paraganglia | 2002 | 166 | 9.22 |
| 73 | Stratakis, CA | The triad of paragangliomas, gastric stromal tumours and pulmonary chondromas (Carney triad), and the dyad of paragangliomas and gastric stromal sarcomas (Carney-Stratakis syndrome): molecular genetics and clinical implications | 2009 | 164 | 14.91 |
| 74 | Blake, MA | Pheochromocytoma: An imaging chameleon | 2004 | 164 | 10.25 |
| 75 | Favier, J | The Warburg Effect Is Genetically Determined in Inherited Pheochromocytomas | 2009 | 163 | 14.82 |
| 76 | Gimenez-Roqueplo, AP | Functional consequences of a SDHB gene mutation in an apparently sporadic pheochromocytoma | 2002 | 162 | 9.00 |
| 77 | Pellitteri, PK | Paragangliomas of the head and neck | 2004 | 160 | 10.00 |
| 78 | Rao, AB | From the archives of the AFIP - Paragangliomas of the head and neck: Radiologic-pathologic correlation | 1999 | 158 | 7.52 |
| 79 | Eisenhofer, G | Plasma methoxytyramine: A novel biomarker of metastatic pheochromocytoma and paraganglioma in relation to established risk factors of tumour size, location and SDHB mutation status | 2012 | 156 | 19.50 |
| 80 | Gill, AJ | Immunohistochemistry for SDHB triages genetic testing of SDHB, SDHC, and SDHD in paraganglioma-pheochromocytoma syndromes | 2010 | 156 | 15.60 |
| 81 | Walther, MM | Clinical and genetic characterization of pheochromocytoma in von Hippel-Lindau families: Comparison with sporadic pheochromocytoma gives insight into natural history of pheochromocytoma | 1999 | 153 | 7.29 |
| 82 | Taieb, D | EANM 2012 guidelines for radionuclide imaging of phaeochromocytoma and paraganglioma | 2012 | 152 | 19.00 |
| 83 | John, H | Pheochromocytomas: Can malignant potential be predicted? | 1999 | 150 | 7.14 |
| 84 | Pasini, B | SDH mutations in tumorigenesis and inherited endocrine tumours: lesson from the phaeochromocytoma-paraganglioma syndromes | 2009 | 147 | 13.36 |
| 85 | Mannelli, M | Pheochromocytoma in Italy: a multicentric retrospective study | 1999 | 147 | 7.00 |
| 86 | Jansen, JC | Estimation of growth rate in patients with head and neck paragangliomas influences the treatment proposal | 2000 | 144 | 7.20 |
| 87 | Bayley, JP | The SDH mutation database: an online resource for succinate dehydrogenase sequence variants involved in pheochromocytoma, paraganglioma and mitochondrial complex II deficiency | 2005 | 140 | 9.33 |
| 88 | Eisenhofer, G | Distinct gene expression profiles in norepinephrine- and epinephrine-producing hereditary and sporadic pheochromocytomas: activation of hypoxia-driven angiogenic pathways in von Hippel-Lindau syndrome | 2004 | 140 | 8.75 |
| 89 | Kinney, MAO | Perioperative management of pheochromocytoma | 2002 | 140 | 7.78 |
| 90 | Persky, MS | Combined endovascular and surgical treatment of head and neck paragangliomas - A team approach | 2002 | 140 | 7.78 |
| 91 | Timmers, HJLM | Staging and Functional Characterization of Pheochromocytoma and Paraganglioma by F-18-Fluorodeoxyglucose (F-18-FDG) Positron Emission Tomography | 2012 | 139 | 17.38 |
| 92 | Lopez-Jimenez, E | Research Resource: Transcriptional Profiling Reveals Different Pseudohypoxic Signatures in SDHB and VHL-Related Pheochromocytomas | 2010 | 139 | 13.90 |
| 93 | Taschner, PEM | Nearly all hereditary paragangliomas in the Netherlands are caused by two founder mutations in the SDHD gene | 2001 | 139 | 7.32 |
| 94 | Plouin, PF | Factors associated with perioperative morbidity and mortality in patients with pheochromocytoma: Analysis of 165 operations at a single center | 2001 | 136 | 7.16 |
| 95 | Tischler, AS | Pheochromocytoma and extra-adrenal paraganglioma - Updates | 2008 | 135 | 11.25 |
| 96 | Pacak, K | 6-[F-18] fluorodopamine positron emission tomographic (PET) scanning for diagnostic localization of pheochromocytoma | 2001 | 135 | 7.11 |
| 97 | Plouin, PF | European Society of Endocrinology Clinical Practice Guideline for long-term follow-up of patients operated on for a phaeochromocytoma or a paraganglioma | 2016 | 133 | 33.25 |
| 98 | Raber, W | Diagnostic efficacy of unconjugated plasma metanephrines for the detection of pheochromocytoma | 2000 | 132 | 6.60 |
| 99 | Eisenhofer, G | Biochemical and clinical manifestations of dopamine-producing paragangliomas: Utility of plasma methoxytyramine | 2005 | 131 | 8.73 |
| 100 | Kudva, YC; Sawka, AM; Young, WF | Clinical review 164 - The laboratory diagnosis of adrenal pheochromocytoma: The Mayo Clinic experience | 2003 | 131 | 7.71 |
